# Cultured Subventricular Zone Progenitor Cells Transduced with Neurogenin-2 Become Mature Glutamatergic Neurons and Integrate into the Dentate Gyrus

**DOI:** 10.1371/journal.pone.0031547

**Published:** 2012-02-14

**Authors:** Xia Chen, Alexandra Lepier, Benedikt Berninger, Aviva M. Tolkovsky, Joe Herbert

**Affiliations:** 1 Cambridge Centre for Brain Repair, Department of Clinical Neurosciences, University of Cambridge, Cambridge, United Kingdom; 2 Department of Physiological Genomics, Institute of Physiology, Ludwig-Maximilians-Universität München, Munich, Germany; 3 Institute of Stem Cell Research, Helmholtz Zentrum München, National Research Centre for Environment and Health, Munich-Neuherberg, Germany; Institut Curie, France

## Abstract

We have previously shown that transplantation of immature DCX+/NeuN+/Prox1+ neurons (found in the neonatal DG), but not undifferentiated neuronal progenitor cells (NPCs) from ventral subventricular zone (SVZ), results in neuronal maturation in vivo within the dentate niche. Here we investigated whether we could enhance the integration of SVZ NPCs by forced expression of the proneural gene Neurogenin 2 (*NEUROG2*). NPCs cultured from neonatal GFP-transgenic rat SVZ for 7 days in a non-differentiating medium were transduced with a retrovirus encoding *NEUROG2* and DsRed or the DsRed reporter gene alone (control). By 3 days post-transduction, the *NEUROG2*-transduced cells maintained in culture contained mostly immature neurons (91% DCX+; 76% NeuN+), whereas the control virus-transduced cells remained largely undifferentiated (30% DCX+; <1% NeuN+). At 6 weeks following transplantation into the DG of adult male rats, there were no neurons among the transplanted cells treated with the control virus but the majority of the *NEUROG2*-transduced DsRed+ SVZ cells became mature neurons (92% NeuN+; DCX-negative). Although the *NEUROG2*-transduced SVZ cells did not express the dentate granule neuron marker Prox1, most of the *NEUROG2*-transduced SVZ cells (78%) expressed the glutamatergic marker Tbr1, suggesting the acquisition of a glutamatergic phenotype. Moreover, some neurons extended dendrites into the molecular layer, grew axons containing Ankyrin G+ axonal initial segments, and projected into the CA3 region, thus resembling mature DG granule neurons. A proportion of *NEUROG2* transduced cells also expressed c-Fos and P-CREB, two markers of neuronal activation. We conclude that *NEUROG2*-transduction is sufficient to promote neuronal maturation and integration of transplanted NPCs from SVZ into the DG.

## Introduction

Neuronal cell loss occurs in several neurological diseases, and transplanting neuronal progenitor cells (NPCs) is one potential remedy [1]. Many studies have explored the ability of transplanted NPCs to generate neurons that can integrate into the host in vivo. Although some neuronal differentiation has been observed, this is generally limited and grafted NPCs mostly remain undifferentiated or become glial cells [2], [3], [4], [5]. Transplantation of committed young neurons instead of undifferentiated NPCs has been found to improve neuronal yield in vivo markedly [5], [6]. This suggests that neuronal differentiation of NPCs before transplantation might be required to increase transplantation success [7].

Neurogenin 2 (*NEUROG2*) is a proneural basic helix-loop-helix (bHLH) transcription factor that is important for the regulation of neurogenesis and the specification of neuronal subtypes during development and in adulthood [8], [9], [10]. Forced expression of *NEUROG2* promotes neuronal differentiation and directs cells towards a glutamatergic neuronal phenotype [11], [12]. Granule neurons in the dentate gyrus (DG) of the hippocampus are glutamatergic; therefore, *NEUROG2*, which plays a key role in dentate neurogenesis during development [13], is an attractive candidate for driving donor NPCs towards the appropriate neuronal identity before transplantation.

Donor NPCs can be isolated from two main regions of the brain: the DG of hippocampus and the subventricular zone (SVZ) of the lateral ventricle. NPCs in these two regions generate different types of neurons: ventral SVZ NPCs migrate to form mostly GABAergic inhibitory interneurons in the olfactory bulb that lack axons, whereas DG NPCs form excitatory glutamatergic neurons in the granule layer that project long axons to the CA3 region of the hippocampus [14]. Increasing evidence suggests that NPCs from different regions of the brain are distinct and are not able to make site-specific neurons in a new location following heterotypic transplantation [5], [15], [16], [17].

Given the neurogenic effect of *NEUROG2* and the benefit of transplanting young neurons compared to NPCs, we asked whether forced expression of *NEUROG2* would increase the population of young neurons among donor NPCs that had been expanded *in vitro*, and lead to improved neuronal differentiation and graft integration in vivo. We focused primarily on SVZ NPCs to determine whether *NEUROG2*-transduced neurons would express a glutamatergic phenotype and thus better serve to reconstitute the damage done to the DG as a result of the transplantation procedure [5].

Cultured NPCs were transduced with a retrovirus encoding *NEUROG2* and DsRed or the DsRed reporter gene alone, and then transplanted into the DG of adult rats. We show that transduction of cultured SVZ NPC with *NEUROG2* was sufficient to increase neuronal differentiation *in vitro* and enhance survival, neuronal yield, and neuronal integration – as glutamatergic neurons that project axons into the CA3 region - in the host hippocampus in vivo. Our results suggest that even heterotransplanted NPCs can integrate into the host DG if an appropriate neuronal fate has been acquired.

## Results

### *NEUROG2* promotes differentiation of cultured SVZ NPCs into glutamatergic neurons

**Figure 1:**
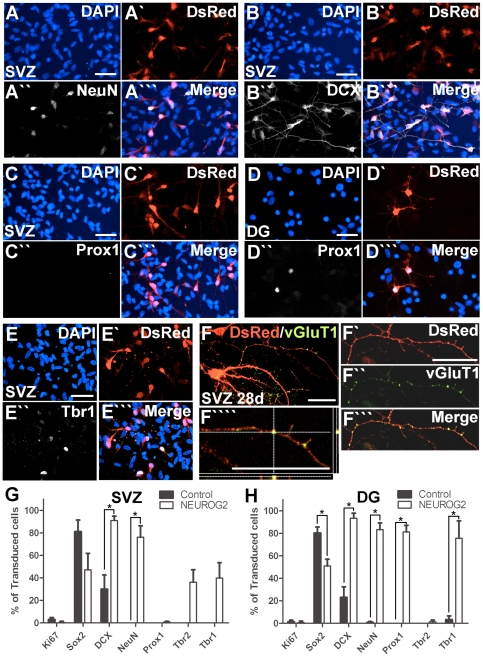
*NEUROG2*-tranduction increases neuronal differentiation of cultured SVZ and DG NPCs *in vitro*. Cells from ventral SVZ and DG of neonatal GFP rats expanded for 7 days as neurospheres in mitogen-containing medium were plated as monolayers and transduced with the *NEUROG2* virus or the control virus. At 3 days post-transduction, the majority of *NEUROG2*-transduced SVZ cells became young neurons, expressing NeuN (A-A′″) and DCX (B-B′″). *NEUROG2*-transduced SVZ cells did not express the DG granule cell marker Prox1 (C-C′″), in contrast to the *NEUROG2*-transduced DG cells (D-D′″). *NEUROG2*-transduced SVZ cells also expressed the T-box transcription factor Tbr1 (E-E′″) and later (28 days) the pre-synaptic vesicular glutamate transporter 1 (vGluT1) (F-F′″1′″), suggesting a shift from their normal GABAergic fate towards the glutamatergic neuronal fate. Percentages of virus-transduced SVZ cells (∼15% of total) (G) and DG cells (∼6% of total) (H) expressing different markers (mean ± SEM, n = 3), *p<0.05. Scale bar = 20 µm.

Following 7 days of expansion as neurospheres, SVZ NPCs from neonatal GFP+ rats were plated down as monolayers and transduced with the retroviruses. At 3 days post-transduction, the survival of the *NEUROG2*-transduced cells and the control virus-transduced cells was similar, based on the numbers of DAPI+ cells per field counted with a ×40 objective (*NEUROG2*, 145±15; Control, 166±17; p>0.05). The rate of transduction was also similar between the *NEUROG2*-transduced and the control virus-transduced cells: about 15% of the SVZ cells expressed the reporter gene DsRed (p>0.05). Among the transduced cells, the majority of the *NEUROG2*-transduced SVZ cells were positive for NeuN (76%, Figs. 1A-A′″, 1G; n≥400 cells) whereas none were observed in control virus-transduced cells (p = 0.004). The majority (91%) of the *NEUROG2*-transduced cells were also positive for the immature neuronal marker DCX, significantly higher than that seen in the controls (30%, p = 0.009) (Figs. 1B-B′″, 1G). Concurrent with neuronal differentiation, the expression of the progenitor cell marker Sox2 in the *NEUROG2*-transduced cells was decreased compared to the controls (47% Vs 81%, p = 0.18, Fig. 1G). Between 36–39% of the *NEUROG2*-transduced SVZ cells were positive for the glutamatergic neuronal markers Tbr2 and Tbr1 (Figs. 1E-E′″, 1G), whereas none were observed in the control, suggesting a shift towards a glutamatergic fate. However, Prox1, a marker of granule neurons of the DG, was not detected in the *NEUROG2*-transduced SVZ cells (Figs. 1C-C′″, 1G).

At 28 days after transduction, 86±11% of RFP+ *NEUROG2*-transduced SVZ cells were positive for NeuN, and 67±8% stained positive for Tbr1 (≥100 cells counted per individual). Furthermore, the expression of the pre-synaptic vesicular glutamate transporter 1 (vGluT1), specific to mature glutamatergic neurons was observed in the *NEUROG2*-transduced SVZ cells (Figs. 1F-F′″1′″). No staining for any of these markers was found in the control virus-transduced SVZ cells. These data indicate that the glutamatergic phenotype continued to develop in the *NEUROG2*-transduced SVZ cells *in vitro* cell autonomously. Expression of Prox1, however, remained absent.

Because we had found previously [5] that DG cells from newborn GFP+ rats cultured for 7 days under proliferative conditions contained few (<10% DCX+/NeuN+) young neurons, and also integrated poorly into the host DG, we asked whether *NEUROG2* could increase the population of these young neurons among the DG progenitors. At 3 days post-transduction, only about 6% of the DG cells expressed DsRed, probably due to lack of viral integration because of their slow rate of proliferation. However, as found in SVZ cells, the majority of the *NEUROG2*-transduced cells expressed NeuN (83%) and DCX (93%) compared to control (23% DCX+, p<0.0001; 1% NeuN+, p<0.0001, Fig. 1H; n≥100 cells). This shift was accompanied by a significant reduction in Sox2+ cells (51% Vs 80%, p = 0.0047, Fig. 1H). The majority (76%) of the *NEUROG2*-transduced DG also expressed Tbr1 (Fig. 1H), consistent with their dorsal telencephalic origin. The expression of the transiently expressed marker Tbr2 was no longer present, suggesting that they had acquired a more a mature glutamatergic phenotype typical of granule neurons. Interestingly, unlike SVZ-derived cells, the majority of the *NEUROG2*-transduced DG cells (81%) were also positive for the DG granule neuronal marker Prox1 (Figs. 1D-D′″, 1H), constituting a substantial overlap with the NeuN+ population. Hence the lack of Prox1 expression in the NEUROG2-transduced SVZ NPCs is not due to an inhibitory effect of *NEUROG2*.

Together, these data suggest that expression of *NEUROG2* enhanced neuronal differentiation of cultured NPCs of SVZ and DG origin *in vitro*, though there was a marked difference in the incidence of Prox1 expression between the DG and SVZ NPCs. We next tested whether transplantation of the *NEUROG2*-transduced cells could lead to better integration of transplanted cells in vivo.

### *NEUROG2*-transduced SVZ cells form mature glutamatergic neurons in the DG after transplantation

Transduced cells were transplanted into the DG of adult male rats on day 3 post transduction and their fate was investigated 6 weeks after transplantation. As we reported previously [5], the suprapyramidal blade of the granule layer in the host DG around the infusion site was damaged by the transplantation procedure and had lost the majority of the resident NeuN+ granule neurons. This damage persisted for up to 6 weeks and was not repaired by endogenous NPCs within this time period.

Six weeks after virus-transduced SVZ cells were transplanted, there remained 4216±838 GFP+ SVZ cells in the control virus-transduced group (8.4±1.7% of injected), similar to the rate of 9.8% we reported previously for non-virally transduced SVZ cells [5]. However, 9719±1505 GFP+ cells of the *NEUROG2*-transduced group (19.4±3.0%) remained in the adult DG. The improvement of graft survival in the *NEUROG2*-transduced group was significant (p = 0.008). The increased survival observed in the *NEUROG2*-tranduced cohort may be due to some fading of dsRed, which we observed in long term grafts after fixation, or to non cell autonomous effects between *NEUROG2*-tranduced and non-transduced cells, as *NEUROG2* expression has been shown to promote intercellular communication via the secretion of the Notch ligand Delta-like 1 during development [18].

**Figure 2:**
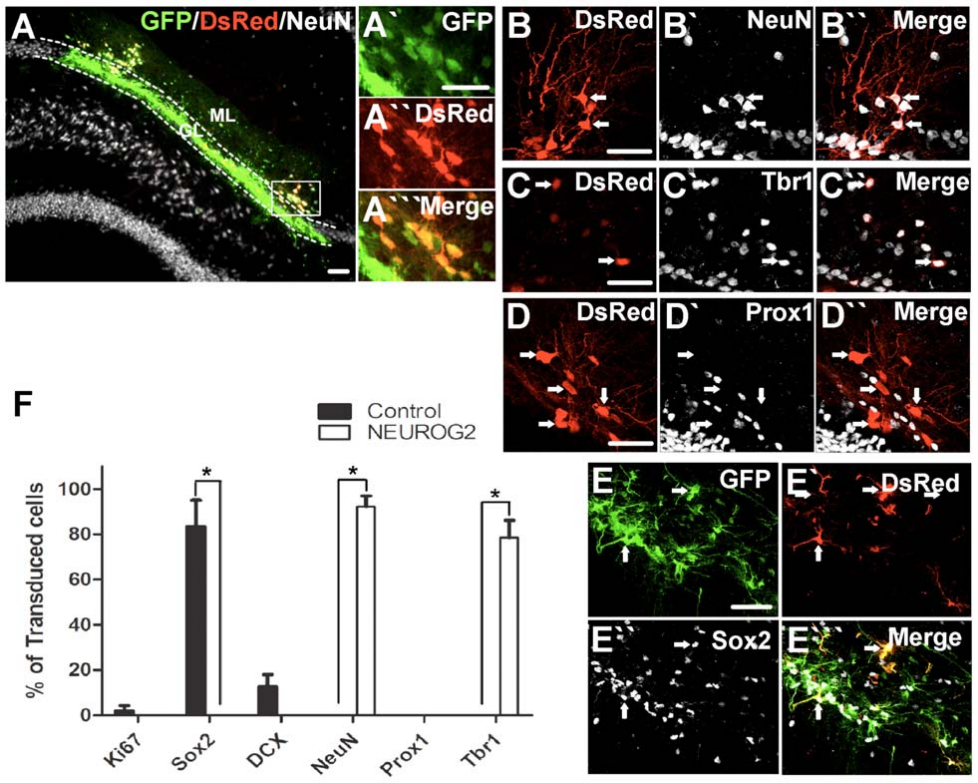
*NEUROG2*-transduced SVZ cells show improved neuronal yield in vivo. On day 3 post-transduction, virus-transduced cells were transplanted into the DG of adult rat and brain tissues were analysed 6 weeks post-transplantation. Example of GFP+ *NEUROG2*-transduced SVZ cells at 6 weeks post-transplantation, highlighting GFP+ cells positive for DsRed (A-A′″). Note that some grafted cells had cell bodies misplaced in the molecular layer (ML) instead of the granule layer (GL). The majority of *NEUROG2*-transduced SVZ cells were positive for NeuN (B-B′″) and Tbr1 (C-C′″) though negative for Prox1 (D-D′″), whereas the control virus-transduced cells were mostly positive for Sox2 (F-F′″). Percentages of virus-transduced SVZ cells expressing different markers at 6 weeks (mean ± SEM, n = 4), *p<0.05 (G). Scale bar = 50 µm.

Among the transduced DsRed+ cells (10% of GFP+ cells in the control; 16% of *NEUROG2*-transduced cells), the majority of the control virus-transduced cells were positive for Sox2 (83%, Figs. 2E-E′″, 2F), ∼12% were DCX+, and none were NeuN+ (n≥140 cells), suggesting that they persisted in an undifferentiated state. The same profile was observed for many of the GFP+/dsRed-negative cells in the *NEUROG2*-transduced cohorts, as noted previously [5]. In contrast, most of the *NEUROG2*-transduced SVZ cells were neurons expressing NeuN (92%, Figs. 2B-B′″, 2F; n≥400 cells). Expression of the immature neuronal marker DCX was no longer detectable, indicating that neuronal maturation had occurred *in vivo* beyond the stage that had been reached three days after transduction and culture *in vitro*. The majority of the *NEUROG2*-transduced cells were also positive for Tbr1 (78%, Figs. 2C-C′″, 2F), an increase compared to transduced neurons at the time of transplantation (∼37%), suggesting that they maintained and/or had newly acquired a glutamatergic phenotype. The expression of Prox1, however, remained absent (Figs. 2D-D′″, 2F).

**Figure 3:**
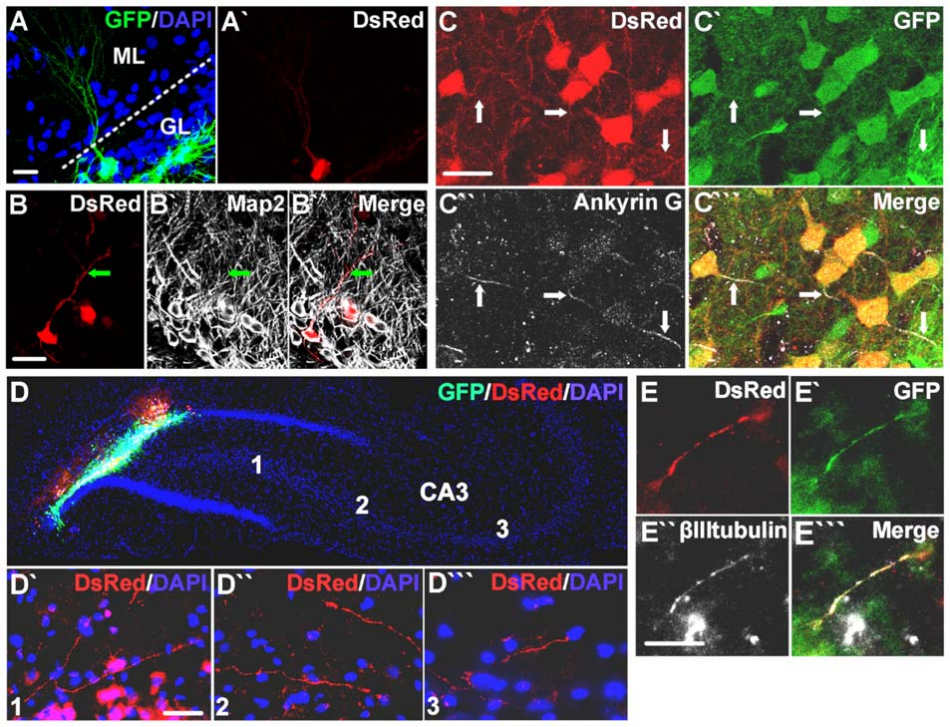
*NEUROG2*-transduced SVZ cells extend dendrites into the molecular layer and innervate the target CA3 in vivo. Similar to endogenous DG granule neurons, the *NEUROG2*-transduced SVZ cells extended elaborate dendrites into the molecular layer (A-A′), which were positive for Map2 (B-B′″). The *NEUROG2*-transduced SVZ cells also expressed the axonal initial segment marker Ankyrin G (C-C′″) and displayed DsRed+ processes in the CA3 (Regions 1–3) (D-D′″). Some of these processes were positive for the neuronal marker βIIItubulin (E-E′″), suggesting the ability of the *NEUROG2*-transduced SVZ neurons to innervate the CA3. Scale bar = 20 µm.

**Figure 4:**
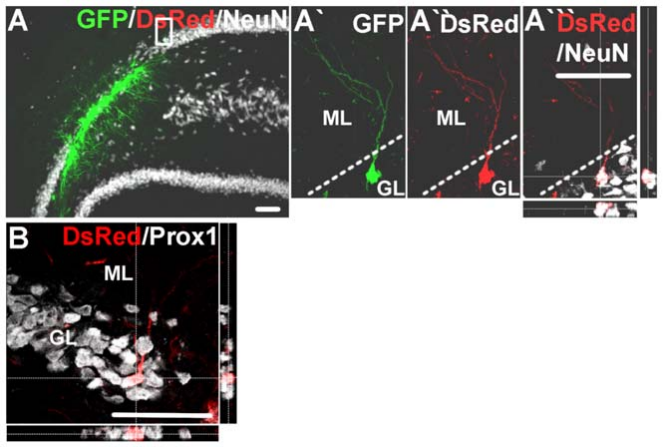
*NEUROG2*-transduced DG cells express Prox1. Example of GFP+ *NEUROG2*-transduced DG cells at 6 weeks post-transplantation (A), with a transduced dsRed+ DG cell positive for NeuN (A′-A′″) and Prox1 (B). Note that the dsRed+ neuron is correctly located in the granule layer of the DG and extends dendrites into the molecular layer, thus morphologically resembling the endogenous DG granule neurons.

The cell bodies of many *NEUROG2*-transduced cells were misplaced towards the molecular layer instead of being in the normal granule layer (Fig. 2A-A′″), a notable discrepancy compared to the position of the endogenous DG granule neurons or transplanted immature DG neurons [5]. Nevertheless, similar to endogenous and immature transplanted DG granule neurons, the majority of the *NEUROG2*-transduced SVZ cells showed extensions of Map2+ dendrites into the molecular layer (98.6% based on 145 RFP+ cells counted) (Figs. 3A-A′, 3B-B′″), which was not observed in the control virus-transduced cells. In addition, many of the *NEUROG2*-transduced SVZ cells also expressed the axonal initial segment marker Ankyrin G (70.1% based on 117 RFP+ cells counted) (Figs. 3C-C′″), indicating the formation of axons. Most importantly, DsRed+ processes from *NEUROG2*-transduced SVZ cells were observed in the target CA3 region (Figs. 3D-D′″) and some were positive for the neuronal marker βIII-tubulin (Figs. 3E-E′″), establishing the ability of the *NEUROG2*-transduced SVZ cells to innervate the CA3 region despite the lack of expression of Prox1. However, *NEUROG2*-transduced cultured DG NPCs did stain positive for NeuN (Fig. 4A′″) and Prox1 (Fig. 4B) 6 weeks after transplantation, whereas control virus-transduced DG cells did not. We also noted that the *NEUROG2*-transduced DG cells were similar to granule neurons in that they extended dendrites into the molecular layer (Figs. 4A-A′″), and had cell bodies positioned appropriately in the granule cell layer. Thus, *NEUROG2* does not prevent expression of Prox1 *per se* even 6 weeks after its transduction.

**Figure 5:**
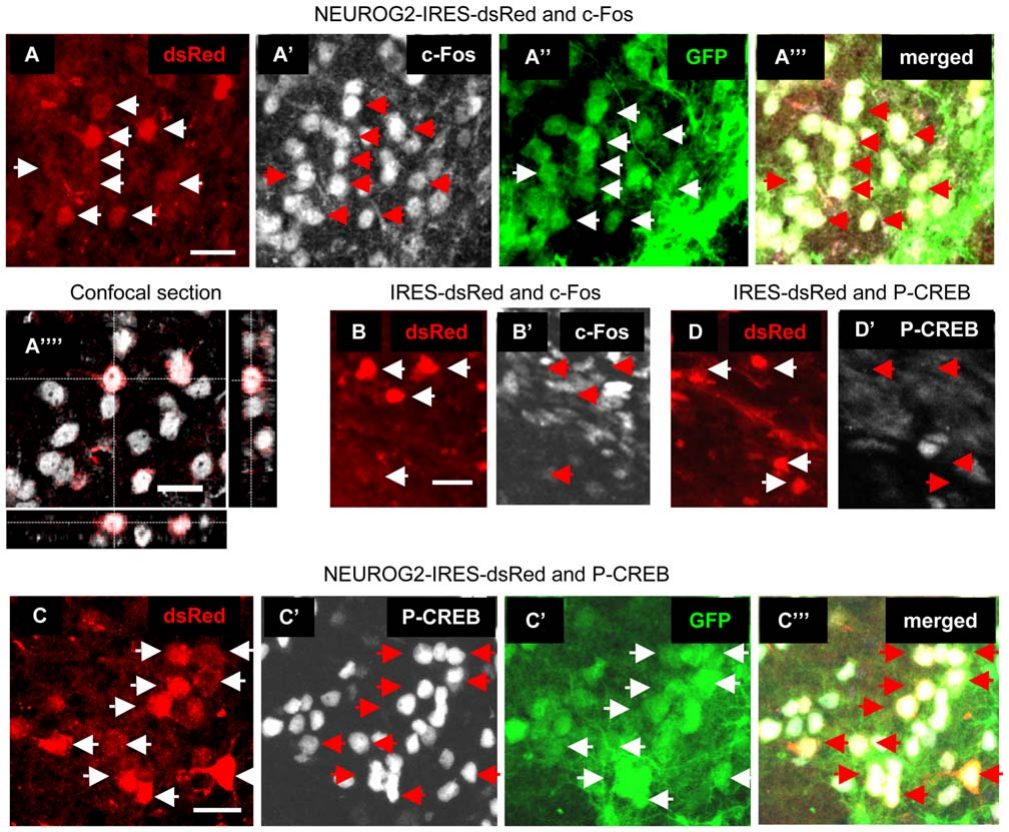
*NEUROG2*-transduced SVZ cells express c-Fos or P-CREB. Brain sections were stained for c-Fos (*NEUROG2*: A-A′″1′″; control: B-B′) or P-CREB (*NEUROG2*: C-C′″1′″; control: D-D′), without enhanced staining for dsRed or GFP. Scale bar = 20 µm.

To investigate whether the *NEUROG2*-transduced neurons were receptive to incoming signals, we stained for c-Fos and Phospho-serine133-CREB (P-CREB), both widely used markers of neuronal activity [19], [20] that are expressed in active DG granule neuron NPCs [21], [22]. Numerous examples of dsRed+ neurons expressing c-Fos and P-CREB were found amongst the cells transduced with *NEUROG2*, but none were observed after expression of the control virus (Fig. 5).

Together, these data show that pre-transplantation transduction with *NEUROG2* improved cell survival and neuronal yield of transplanted SVZ cells. *NEUROG2*-transduced SVZ neurons showed a shift towards a glutamatergic fate, extended dendrites into the molecular layer, innervated the CA3 region, and expressed c-Fos and P-CREB thus morphologically resembling endogenous DG granule neurons, despite remaining negative for Prox1 in vivo.

## Discussion

Previously we showed that cultured NPCs derived from neonatal rat SVZ had a limited ability to produce neurons when transplanted into the DG of the adult rat, whereas freshly isolated neonatal DG cells that contained a high proportion of young neurons survived better and robustly reconstituted a DG that had been damaged as a result of the transplantation procedure. Here, we asked to what extent we could enhance the population of young neurons among the SVZ NPCs prior to transplantation through expression of the pro-neural gene *NEUROG2* and thereby overcome the failure of the SVZ cells to reconstitute the host DG.

We show that transduction of cultured NPCs with *NEUROG2 in vitro* was sufficient to drive the production of young neurons positive for DCX and NeuN within 3 days. This is consistent with the pro-neural effect of *NEUROG2* in a range of cells including glial progenitors and non-neuronal cells [8], [12], [23], [24]. Although virus-transduced control cells also contained ∼20% DCX+ cells, the incidence of expression of the more mature neuronal marker NeuN was <1%, implying an additional role for *NEUROG2* in neuronal maturation [25]. *NEUROG2*-transduced ventral SVZ cells expressed the dorsal telencephalic markers Tbr2/Tbr1 and later (28 days) the glutamatergic neuronal marker vGluT1 *in vitro*, suggesting a switch from the GABAergic fate towards a glutamatergic fate more typical of the dorsal telencephalus. This agrees with the postulated role of *NEUROG2* in specifying a glutamatergic neuronal subtype [8], [9], [11], [26]. However, the *NEUROG2*-transduced SVZ cells did not express the DG granule cell transcription factor (and marker) Prox1 after either 3 or 28 days *in vitro*, unlike the *NEUROG2*-transduced DG cells, suggesting that either the expression of Prox1 is not directly driven by *NEUROG2* or that additional factors are required for its induction [27], [28].

Given the importance of Prox1 for DG development and neurogenesis [29], [30], we next examined whether Prox1-negative SVZ cells were able to integrate into the host DG. After six weeks, we found a high incidence of *NEUROG2*-transduced SVZ NeuN+/Tbr1+ neurons that projected dendrites into the molecular layer, several with axons expressing the axon initial segment marker Ankyrin G, and some βIIItubulin+ axons projecting into the CA3 region. The *NEUROG2*-transduced SVZ cells displayed a higher survival rate than control virus-transduced cells, supporting a proposed role for *NEUROG2* in promoting graft survival [7]. This may be partly non-cell autonomous [18], given that cells that appeared dsRed-negative were also found in higher abundance than control transduced cells. However, although there were ∼2000 *NEUROG2*-transduced SVZ cells, the number of projections to the CA3 region was much lower than the 20% predicted based on the number of projections from ∼10.000 primary DG NPCs transplanted for 6 weeks [5]. It may be that the intrinsic ability of the SVZ cells to project to CA3, not a natural target, is reduced compared to DG-derived cells. Another reason may be that many were displaced within the molecular layer, whereas primary DG cell transplants were almost exclusively located in the granule layer [5], as were the *NEUROG*2-transduced DG cells. Thus, SVZ cells may not be able to respond completely to local guidance signals such as reelin [31]. Nevertheless, it seems likely that the DG exerts some environmental influence on the *NEUROG2*-transduced transplanted SVZ cells since most expressed the axon initial segment marker Ankyrin G, whereas the *NEUROG2*-transduced SVZ cells that were cultured *in vitro* for 28 days did not develop axons.

Although positive for Tbr1, *NEUROG2*-transduced SVZ neurons remained negative for Prox1 six weeks after grafting, despite transplantation into a DG environment rich with Prox-1+ neurons. *NEUROG2*-transduction is unlikely to prevent cells from expressing Prox1 since *NEUROG2*-transduced DG NPCs were induced to become NeuN+ and Prox1+, and resembled endogenous DG granule neurons morphologically. Intrinsic differences in SVZ and DG NPCs might explain this difference, as DG NPCs were positive for Prox1 but already negative for Tbr1, whereas SVZ cells were negative for Prox1 but positive for both Tbr1 and Tbr2. It is possible that the *NEUROG2*-transduced ventral SVZ cells became olfactory bulb-like glutamatergic neurons in the host DG, as described recently [10], [26], [32]. However, over 80% of the *NEUROG2*+ neurons generated in our study expressed NeuN whereas <0.2% of SVZ-derived *NEUROG2*+ olfactory bulb neurons were NeuN+ [26]. New markers are needed to differentiate all the subtypes of glutamatergic neurons [33]. Effecting a complete switch of progenitor cell identity after heterotypic transplantation is rare, and depends on the recipient site as well as the donor identity [5], [15], [17], [34], [35]. It remains to be determined whether longer periods of culture of SVZ NPCs would enable them to express Prox1 following *NEUROG2*-transduction.

Interestingly, however, despite being Prox1-negative, the *NEUROG2*-transduced SVZ neurons were still able to extend dendrites into the molecular layer, showed some innervation of CA3, and were activated to express c-Fos and P-CREB, indicating that they are receptive to incoming signals. This raises the question whether the permanent expression of Prox1 is necessary for the generation of DG granule-like neurons by non-DG NPCs such as SVZ NPCs. In preliminary experiments, we found that expression of Prox1 was achieved *in vitro* by addition of activin A for 3 days, similar to that in the intact hippocampus [28], but the induction was reversed upon activin A withdrawal. Whether expression of Prox1 through viral transduction or by adding inducing factors, e.g. wnt [27] will improve the integration of SVZ cells and whether they achieve functionality similar to endogenous granule neurons remains to be tested.

Overall, we conclude that pre-transplantation neuronal differentiation of SVZ NPCs by expression of *NEUROG2* improves graft survival, neuronal yield and graft integration. Given that endogenous NPCs failed to repopulate the injured DG, and that cultured SVZ provide a rich source of NPCs, converting them into appropriate young neurons by transduction of *NEUROG2* neurons may be one way of repairing the damaged DG. Our results reinforce the benefits of transplanting young neurons over undifferentiated cells and define the role of the pro-neural gene *NEUROG2* in promoting this phenotype.

## Materials and Methods

### Cell culture of NPCs and viral transduction

Donors were postnatal (P2–P4) rats expressing green fluorescent protein (GFP) ubiquitously under the control of the β–actin promoter. The brains were removed and placed in cold HBSS. The DG and the ventral region of SVZ were then carefully dissected out under a light microscope. Neurosphere culture of primary NPCs followed the protocol we described previously [5]. After 7 days in culture, neurospheres were harvested, digested in Accutase solution at 37°C for 10 min and mechanically dissociated into a single cell suspension. Cells were plated onto flasks or glass coverslips precoated with laminin (10 µg/ml) at a density of ∼40,000 cells per cm^2^ in growth medium containing FGF-2 and EGF. Transduction with pCAG-*NEUROG2*-IRES-DsRed or DsRedExpress2 or control (pCAG-IRES-DsRed or DsRedExpress2) retroviruses followed the protocol described previously [36]. Virus (∼60 particles per cell) was added 2–3 h after plating and 24 hr later, medium was replaced with differentiation medium (Neurobasal A containing 1% L-glutamine, 2% B-27 supplement and 1% penicillin-streptomycin-amphotericin mixture). Every 3 days, half of the medium was replaced with fresh differentiation medium. Cells were fixed in 0.1 M phosphate buffered saline (PBS) containing 4% paraformaldehyde (PFA) for 10 min followed by washing with 0.1 M PBS.

### Transplantation

Three days after onset of retroviral transduction, cells were transplanted into the DG of adult male rats (Sprague-Dawley; weight 250–300 g). All procedures were approved by the Cambridge local ethical committee and carried out under UK Home Office licence guidelines. Five to six animals were transplanted with each cell type. On the day of surgery, donor cells were harvested by incubating cell monolayers in Accutase solution at 37°C for 5 min. Detached cells were spun and re-suspended in DMEM at a density of 50,000 cells/µl. Cell transplantation followed the protocol we described previously [5] using the following coordinates: 3.6 mm posterior to bregma, 1.4 mm from the midline, and 4.0 mm below the dural surface. After a survival time of 6 weeks, recipients were perfused with 4% PFA and brains were further fixed in 4% PFA overnight before transfer to 30% sucrose. Coronal sections (25 µm) were cut using a freezing microtome starting from 1.5 mm rostral to the transplantation site to 1.6 mm posterior to it. Every 6^th^ section was checked for the presence of transplanted GFP positive cells by fluorescence microscopy.

### Immunostaining

Cells or tissue sections were stained using the following primary antibodies: anti-GFAP (C9205), anti-GFP (A11122), anti-Prox1 (P0089, all from Sigma), anti-RFP (22904, Rockland), anti-NeuN (MAB377, Chemicon), anti-Map2 (ab11268, Abcam), anti-βIIItubulin (T8660, Sigma), anti-Ki67 (VP-RM04, Vector), and anti-DCX (sc8066), anti-Sox2 (sc17320, both from Santa Cruz), were all used at 1∶500. Anti-Ankyrin G 1∶100 (N106/36, Neuromab), anti-S100β 1∶300 (ab52642), anti-GFP-FITC 1∶500 (ab6662, both from Abcam), anti-vGluT1 1∶1000 (ab5905, Millipore), anti-c-Fos 1∶200 (PC38, Ab-5, Calbiochem), anti-phospho-serine133-CREB 1∶200 (06-519, Upstate). DAPI (10 µg/ml, D9542, Sigma) was used to identify nuclei. Secondary AlexaFluor®-conjugated antibodies (1∶500) were from Invitrogen. No non-specific binding was observed when cells or tissues were incubated with secondary antibodies only. DAB staining followed the procedure described in [5].

### Quantification

To characterise cells *in vitro* before transplantation, three coverslips from three separate experiments were stained for each marker. On each coverslip, 5–7 images were acquired 1 mm apart using a fluorescence microscope (Leica) with a ×40 oil immersion (NA 1.25) objective. Images were opened in LAS AF Lite software and the number of marker-positive cells was counted. Co-localization was determined by overlapping different channels and merging images. To characterise cells in-vivo, only animals that had the majority of the transplanted cells in the suprapyramidal layer were included for data analysis. Every 12^th^ section was processed for staining. To determine transplant survival, tissue sections stained with anti-GFP antibody were processed for DAB reaction. The number of GFP+ cells was counted using the optical fractionator method with the Stereo Investigator programme (MBF Bioscience) as described previously [5]. The percentage of marker-positive cells was calculated by the dividing the number of marker-positive GFP+ or DsRed+ cells over the total number of GFP+ or DsRed+ cells respectively.

### Statistics

Data are presented as mean ± SEM. Unpaired student t-tests were used for between group comparisons. One sample t-tests were used when all values in one group were equal to zero. Probability levels p<0.05 were considered to be significant.
